# Effect of phenolic extracts from different extra-virgin olive oil varieties on osteoblast-like cells

**DOI:** 10.1371/journal.pone.0196530

**Published:** 2018-04-26

**Authors:** Lucía Melguizo-Rodríguez, Javier Ramos-Torrecillas, Francisco Javier Manzano-Moreno, Rebeca Illescas-Montes, Ana Rivas, Concepción Ruiz, Elvira De Luna-Bertos, Olga García-Martínez

**Affiliations:** 1 Biomedical Group (BIO277), Department of Nursing, Faculty of Health Sciences, University of Granada, Granada, Spain; 2 Instituto Investigación Biosanitaria, ibs.Granada, Granada, Spain; 3 Biomedical Group (BIO277), Department of Stomatology, School of Dentistry, University of Granada, Granada, Spain; 4 Biomedical Group (BIO277), Department of Nursing, Faculty of Health Sciences (Melilla), University of Granada, Melilla, Spain; 5 AGR-255 Group, Department of Nutrition and Food Sciences, Faculty of Pharmacy, University of Granada, Granada, Spain; 6 Institute of Neuroscience Federico Olóriz, University of Granada, Armilla, Granada, Spain; University of Palermo, ITALY

## Abstract

The reported incidence of osteoporosis is lower in countries in which the Mediterranean diet predominates, and this apparent relationship may be mediated by the phenolic compounds present in olive oil. The objective of this study was to determine the effect of phenolic extracts from different varieties of extra-virgin olive oil (Picual, Arbequina, Picudo, and Hojiblanca) on the differentiation, antigenic expression, and phagocytic capacity of osteoblast-like MG-63 cells. At 24 h of treatment a significant increase in phosphatase alkaline activity and significant reductions in CD54, CD80, and HLA-DR expression and in phagocytic activity were observed in comparison to untreated controls. The *in vitro* study performed has demonstrated that phenolic compounds from different extra virgin olive oil varieties can modulate different parameters related to osteoblast differentiation and function.

## Introduction

Osteoporosis affects elderly men and women world-wide and is becoming increasingly frequent as people live longer. Non-pharmacologic approaches to bone health improvement include dietary measures, notably adherence to a diet rich in protein, fruits, and vegetables, such as the Mediterranean diet [1]. Olive oil is considered the main fat source in this dietary model and is highly appreciated for its characteristics and nutritive properties. The biological properties of phenolic compounds in extra virgin olive oil (EVOO) are well documented [2] and are likely to account for its positive effects. These compounds have been found to reduce the risk of coronary disease [3], cancer [4,5], atherosclerosis, and stroke [6], among other diseases. The consumption of olive oil and olive polyphenols has also been found to prevent bone mass loss in animal and cell models, which may be attributable to their induction of a reduction in oxidative stress and inflammation, although published results have not been wholly consistent [7–11]. Our group previously demonstrated that human MG-63 osteosarcoma cell growth and differentiation were induced by EVOO [9,12]. The amount of polyphenols varies among EVOOs depending on the geographic area [13–16], agro-climatic conditions, degree of fruit ripeness, and the oil extraction process itself [11,17–19]. Thus, oil from olives collected at the end of the harvest period was found to have a smaller effect on osteoblast proliferation due to its lesser total phenolic content [20].

Bone is a complex tissue under continuous remodeling, and osteoblasts are the cells responsible for bone formation and regeneration. They have also been attributed with immunological functions, including the expression of markers associated with antigen-presenting capacity (CD54, CD80, CD86, HLA-DR), allogeneic T-cell stimulation, phagocytic activity, and cytokine synthesis [21–23]. The presence of certain growth factors, such as transforming growth factor β1 (TGF-β1), fibroblast growth factor (FGF-bb), and platelet-derived growth factor (PDGF), has been found to decrease the expression of antigen presentation markers in osteoblasts [24]. Olive oil phenolic compounds modulate these parameters on the osteoblast [12], however, it is unknown whether phenolic extracts, which are rich in these compounds, preserve this functional capacity.

The need of studies that clarify the effect of Mediterranean diet on bone health, in order to establish the role of this diet in the prevention of osteoporosis has been addressed by many authors. In addition to being the predominant source of fat in the Mediterranean diet, virgin olive oil is a source of phenolic compounds with biological properties. However, despite the myriad of potential health benefits of olive oil phenolic compounds, there´s no many data published on the possible effects of olive oil phenolic fraction on bone maturation.

Despite of *in vitro* studies limitations our results provide new knowledge about molecular mechanism of action of EVOO phenolic extracts on bone cells, which support the epidemiological evidences which suggest that EVOO could be useful to enhance bone health and prevent osteoporosis.

The objective of this study was to determine the *in vitro* effect of the phenolic fraction of different EVOO varieties on the differentiation, antigenic expression, and phagocytic activity of osteoblast-like MG-63 cells. The MG63 cell line is commonly used as an osteoblast model because they share the same characteristics.

## Material and methods

### Olives

Olives were hand-picked at the beginning of the harvest from ten 27-year-old olive trees of Picual and Hojiblanca cultivars and ten 16-year-old olive trees of Picual, Arbequina, Picudo, and Hojiblanca cultivars that had been grown, spaced 12 x 12 m^2^, in the experimental farm of the Agricultural Research Training Centre in Cabra (Córdoba, Southern Spain). The olive ripening index was determined according to the international olive oil council (IOOC) and based on the olive skin and pulp color [25]. Only healthy fruits with no kind of infection or physical damage were selected for study.

### Oil samples

EVOO samples were obtained using an Abencor analyzer (Abengoa S.A., Seville, Spain), which reproduces the industrial process at laboratory scale and consists of three basic elements: hammer mill, thermobeater, and pulp centrifuge [26]. The EVOO samples obtained were decanted and stored in amber glass bottles at 4°C in darkness without headspace until analysis.

### Sample preparation

The phenolic fraction of the EVOO extracts studied has been previously characterized by García-Martínez et al. (2016) [20]. This fraction was isolated using the method proposed by the IOOC [25]. Briefly, this analytical methodology combined olive oil extraction with methanol/water (80/20), ultrasonic bath for 15 min at ambient temperature, and centrifugation at 5000 rpm for 25 min. Next, an aliquot of the supernatant phase was filtered through a 5-mL plastic syringe using a Millex®-HV PVDF 0.45 μm filter (Millipore Corp, Billerica, MA, USA). Extractions were replicated three times, and phenolic extracts were stored at -20°C until analysis.

### Cell culture

The human osteosarcoma cell line MG-63 was purchased from American Type Cultures Collection (ATCC, Manassas, VA) and maintained in Dulbecco’s Modified Eagle Medium (DMEM; Invitrogen Gibco Cell Culture Products, Carlsbad, CA) with 100 IU/mL penicillin (Lab Roger SA, Barcelona, Spain), 50 μg/mL gentamicin (Braun Medical SA, Jaen, Spain), 2.5 μg/mL amphotericin B (Sigma, St Louis, MO, USA), 1% glutamine (Sigma, St Louis, MO, USA), and 2% HEPES (Sigma, St Louis, MO, USA) supplemented with 10% fetal bovine serum (FBS) (Gibco, Paisley, UK) as described by Díaz-Rodríguez et al. (2009) [27]. Cultures were kept at 37°C in a humidified atmosphere of 95% air and 5% CO_2_. Cells were detached from the culture flask with a solution of 0.05% Trypsin (Sigma, St Louis, MO, USA) and 0.02% ethylenediaminetetraacetic acid (EDTA) (Sigma, St Louis, MO, USA) and were then washed and suspended in complete culture medium with 10% FBS. Before each experiment, all cells were grown in estrogen-free media (DMEM without red phenol) for at least 24 h.

### Cell treatment with oil extracts

Cells were seeded in 24-well plates at 2×10^4^ cells/mL per well in estrogen-free culture medium without FBS and cultured at 37°C in a humidified atmosphere of 95% air and 5% CO_2_ for 24 h. The medium was then replaced with DMEM containing prepared phenolic extracts dissolved in fresh culture medium at a concentration of 0.00001%. All experiments included cells incubated under the same conditions without treatment (controls) and cells incubated with 0.001% methanol as internal control. Three separate experiments were carried out for each treatment, and every experiment was performed in triplicate.

### Alkaline phosphatase activity

The effect on osteoblast-like cell differentiation was assessed by evaluating the alkaline phosphatase (ALP) activity of MG-63 cells cultured for six days in osteogenic medium [28] and then treated with olive oil phenolic extracts at doses of 10^-6^M for 48 h. ALP activity was quantified using a colorimetric assay (Diagnostic kit 104-LL, Sigma, St. Louis, MO, USA) following Sandrini et al [29]. This assay measures the conversion by ALP enzyme of the colorless substrate p-nitrophenyl phosphate to the yellow product p-nitrophenol, with the color change rate corresponding to the amount of enzyme present in the solution. Standard solutions of p-nitrophenol (0–250 μM) were prepared from dilutions of a 1,000 μM stock solution and assayed in parallel. Cells were seeded into 24-well plates at 1×10^4^ cells/mL per well and cultured in the osteogenic medium under standard conditions for 7 days. The culture medium was then replaced by a fresh medium, and cells were treated with 10^−6^ M of the olive oil phenolic extracts under study, followed by their culture for 48 h under standard conditions. Untreated cells were used as control group. Finally, the cells were lysed with 0.1% (v/v) Triton X-100 at 37°C. Samples were then centrifuged at 1,500 rpm, and the supernatants were stored at −70°C until their use. ALP activity was determined by using p-nitrophenol phosphate as substrate, adding the cell lysate solution (50 μL) to 50 μL of ALP substrate (Sigma, St Louis, MO, USA) and incubating the resulting solution at 37°C for 45 min in darkness. The enzymatic reaction was stopped by adding 50 μL of 0.1 M NaOH, and absorbance was then measured at 405 nm with a spectrophotometer (Biotek EL×800). The total protein content was estimated with a protein assay kit from Bio-Rad Laboratories (Nazareth-Eke, Belgium) using Bradford’s method. All samples were run in triplicate, and specific ALP activity was expressed in U/mg cellular protein.

### Antigenic phenotype

Antigenic phenotype was studied by flow cytometry at 24 h of culture after treatment with olive oil phenolic extracts. Untreated cells were used as controls. Cells were then detached from the cultured flask by treatment with 0.4% (*w*/*v*) EDTA solution, washed, and suspended in PBS at 2×10^4^ cells/mL. Cells were labeled by direct staining with the monoclonal antibodies (MAbs) CD54, CD80, CD86, and HLA-DR (CD54/IOL1b, CD80, CD86, and OKDR, respectively, from Invitrogen Corp, Carlsbad, CA, USA). Aliquots of 100 μL cell suspension were incubated with 10 μL of the appropriate MAb for 30 min at 4°C in darkness. Cells were washed, suspended in 1 mL PBS, and immediately analyzed in a flow cytometer with diode laser (FASC Canton II, SE Becton Dickinson, Palo Alto, CA, USA) at a wavelength of 488 nm to determine the percentage of fluorescent cells. Untreated cells were used as controls. The percentage of antibody-positive cells was calculated from counts of 2,000–3,000 cells. At least three experiments were run for each antigen in all cultures.

### Phagocytic activity

Phagocytic activity was studied by flow cytometry at 24 h of culture after treatment with olive oil phenolic extracts. Untreated cells served as controls. Human MG-63 osteosarcoma cells were detached from the culture flask by treatment with 0.04% EDTA solution, washed, and then suspended in a complete culture medium with 10% FBS at 1×10^6^ cells/mL. Cells were labeled by direct staining with labeled latex beads, incubating a 100-μL cell suspension with 200-μL carboxylated FICT-labeled latex beads with a diameter of 2 μm (Sigma, St Louis) for 30 min at 37°C in darkness. Cells were washed, suspended in 1 mL PBS, and immediately analyzed in a flow cytometer (FASC Canton II, SE Becton Dickinson, PaloAlto, CA). Control assays were carried out at 4°C. Results were expressed as the percentage of cells positive for phagocytosis.

### Statistical analysis

Data were expressed as means ± standard deviation (SD). Analysis of variance (ANOVA) was performed to examine the effects on ALP synthesis of treatment with the different olive oil phenolic extracts in comparison to controls. After Shapiro-Wilk test results showed that study variables were normally distributed, the Student’s t-test was used to compare antigenic profile and phagocytic activity between treated and control groups. SPSS 22.0 (IBM, Chicago, IL) was used for the data analysis. P < 0.05 was considered significant in all tests.

## Results

### Effects of olive oil phenolic extracts on ALP of MG-63 osteoblast cells

**Fig 1:**
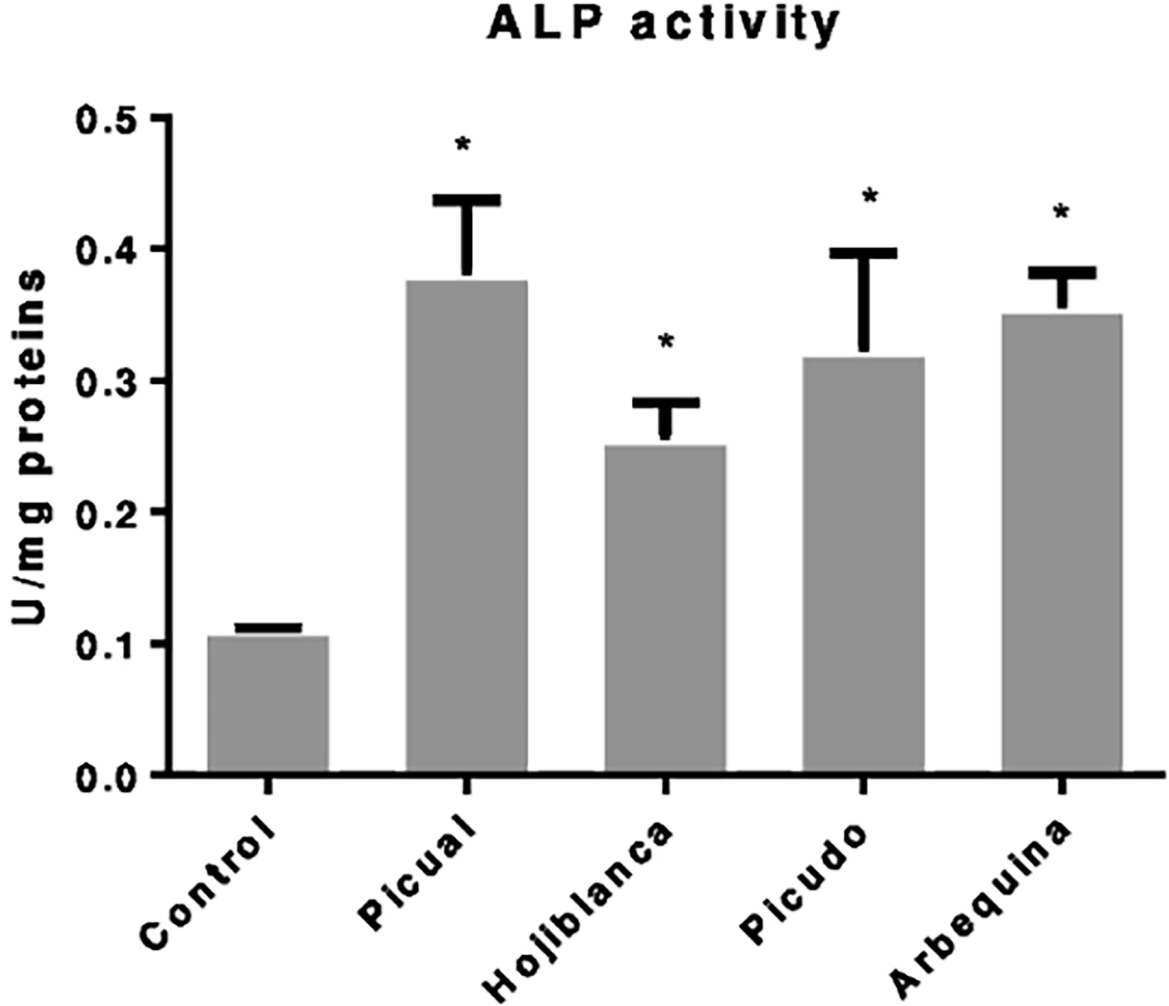
Alkaline phosphatase activity in MG-63 cell line after 48h of olive oil phenolic extract (Picual, Hojiblanca, Picudo and Arbequina) at doses of 10^-6^M. **Data are expressed as U/mg proteins.** *Significant difference (p<0.05).

The ALP activity of osteoblast-like cells cultured in osteogenic medium was significantly increased (p<0.05) by treatment with each EVOO phenolic extract studied in comparison to untreated cells (Fig 1). The greatest effect was obtained with Picual, which produced a 71.05% increase in ALP activity *versus* untreated cells (p = 0.015), followed by Arbequina (69.01% increase vs. controls, p = 0.000).

### Effect of olive oil phenolic extracts on antigenic phenotype of MG-63 cell line

**Fig 2:**
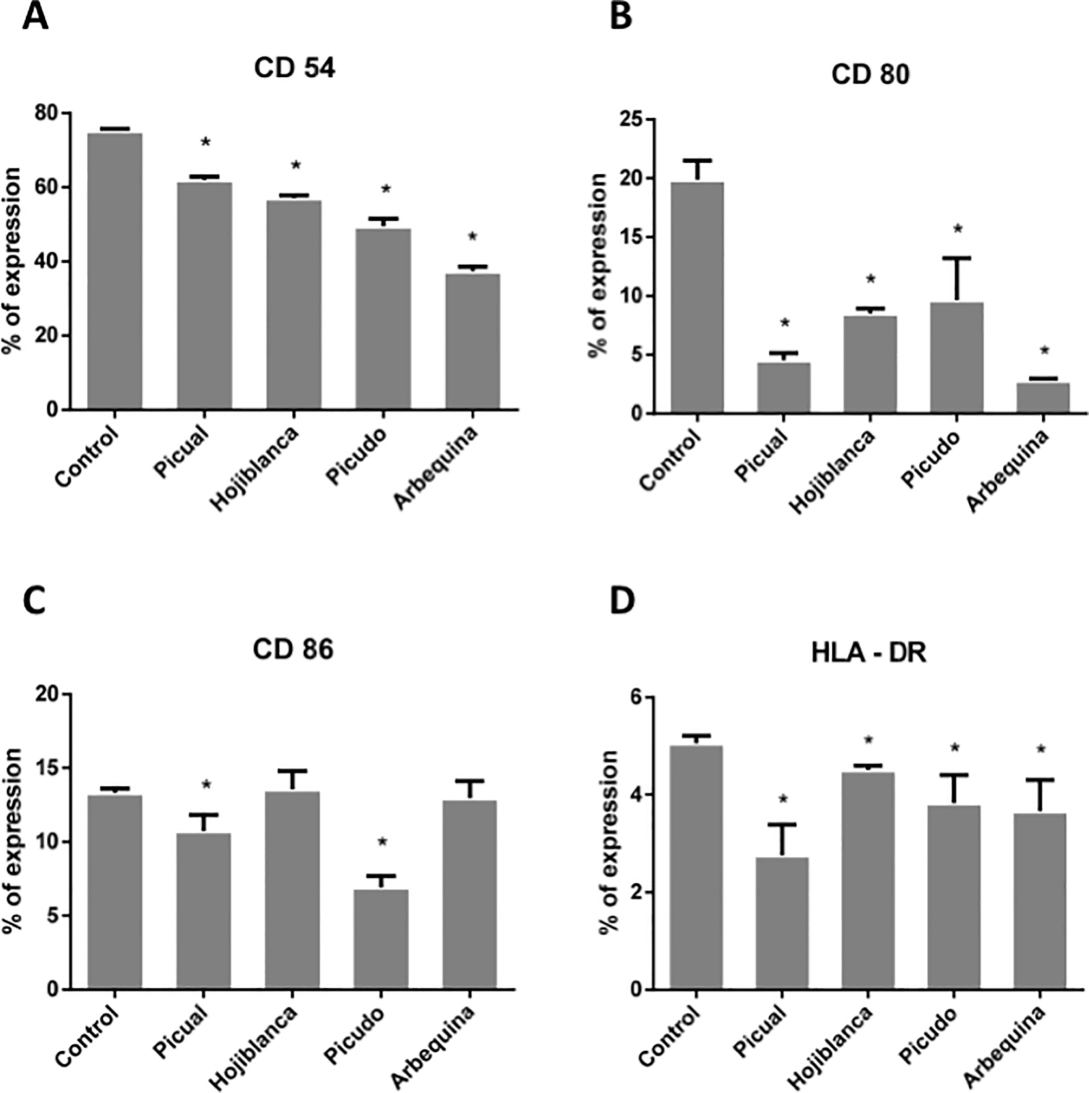
Percentage expression of antigens CD54, CD80, CD86 and HLA-DR in MG-63 cell line after 24 h of treatment with olive oil phenolic extracts. *Significant difference (p<0.05).

Fig 2 depicts the flow cytometry results, showing a significantly reduced expression of CD54, CD80, CD86, and HLA-DR membrane antigens *versus* controls in MG-63 cells cultured for 24 h after each olive oil extract treatment with the exception of Hojiblanca or Arbequina varieties, for which no significant difference was found in the expression of CD86 between control and treated cells.

### Effect of olive oil phenolic extracts on the phagocytic activity of MG-63 cells

**Fig 3:**
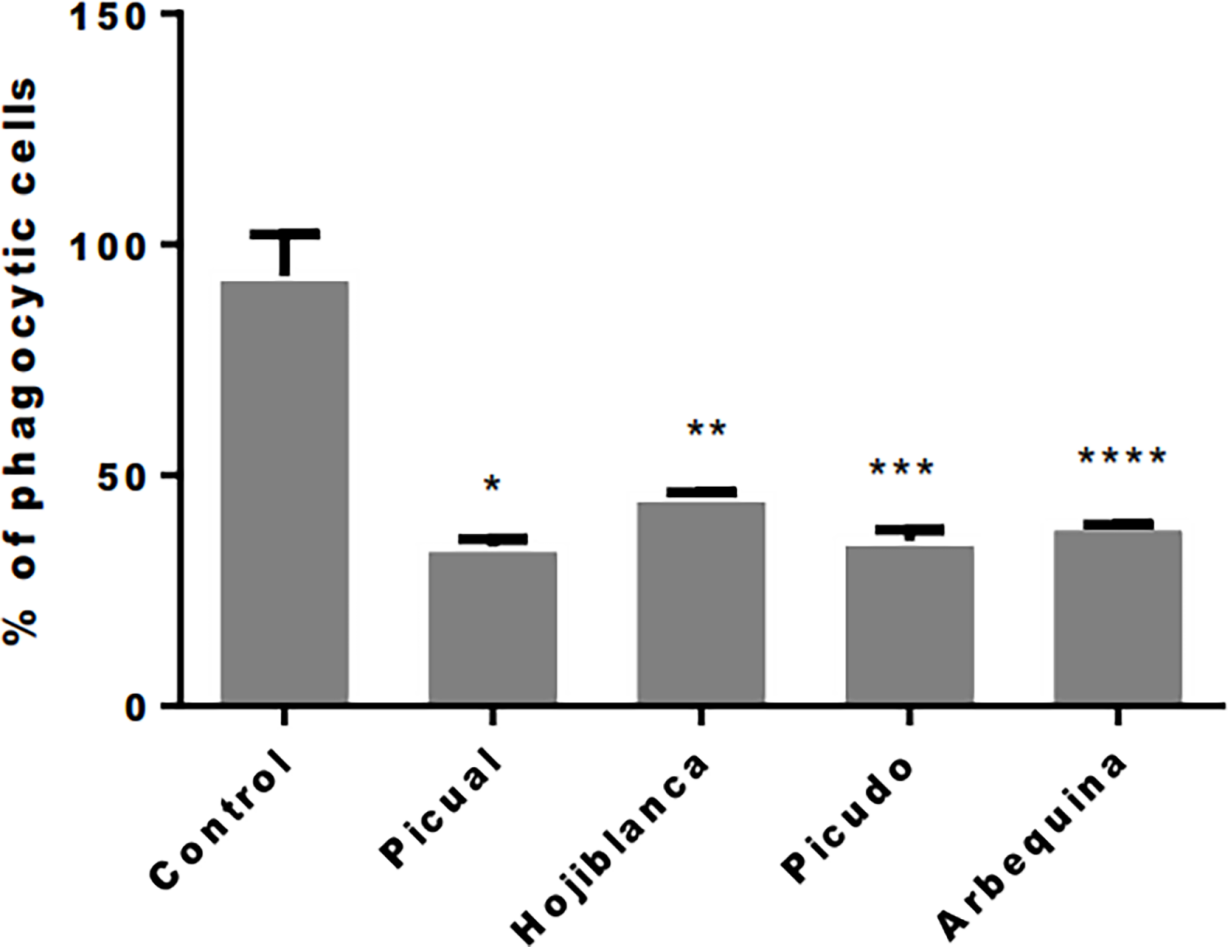
Flow cytometry results for percentage of phagocytic cells in MG-63 cell line at 24 h of olive oil phenolic extracts treatment. **Treatment groups: Picual 10**^**-6**^**M, Hojiblanca 10**^**-6**^**M, Picudo 10**^**-6**^**M, Arbequina 10**^**-6**^**M. Control group: not treated cell.** * p = 0.007; ** p = 0.011; *** p = 0.006; **** p = 0.009.

Fig 3 shows that the phagocytic activity of MG-63 cells was significantly reduced *versus* untreated controls in cells cultured for 24 h after treatment with EVOO phenolic extracts (p<0.05). The percentage reduction *versus* controls ranged from 62.8% with Picual to 51.3% with Hojiblanca.

## Discussion

The main finding of this *in vitro* study is that phenolic extracts from different EVOO varieties can modulate parameters related to osteoblast differentiation.

The expression of CD54, CD80, and HLA-DR on osteoblast-like cells was reduced by treatment with phenolic extracts from every EVOO variety studied. Expression of these antigen presentation markers is known to be modified by the presence of bacterial lipopolysaccharide (LPS), cytokines, growth factors, platelet-rich plasma, and certain pharmaceuticals, among other substances [24,30,31]. Thus, *in vitro* treatment with TGF-β1 of human osteoblasts obtained by primary culture from bone samples was found to significantly reduce their expression of CD54 and also CD86, although it had no significant effect on their expression of CD80 or HLA-DR [24]. In the latter study, the expression of CD54, CD80, CD86, or HLA-DR was not modulated by treatment with FGFb, PDGF-BB, or IL-2 but was significantly increased by treatment with IL-1β, IFNγ, or LPS. Ruiz et al [22] also observed that IL1, TNFβ1, or LPS treatment modified the expression of IL-4, IL-12, IL-15, IL-18, and IFNγ on osteoblasts and suggested that their functional capacity is modulated during differentiation and maturation, with a gain in bone-forming function at the expense of their immunological function. The present findings support this proposition, suggesting that phenolic extracts of EVOOs contribute to the bone-forming function of osteoblasts. Variations observed in the effects of extracts from different varieties are consistent with a previous report on their differential impact on cell proliferation [20]. Thus, Arbequina, which produced the greatest reduction in the expression of cell differentiation markers, had shown the lowest effect on proliferative capacity. This is because the proliferative capacity of the cells decreases with increased maturation/differentiation.

Osteoporosis is a dysregulation of bone remodeling in which the bone resorption rate is greater than the bone formation rate, resulting in bone loss [32]. Etiologic factors implicated in this disease include inflammation and oxidative stress [33], while proinflammatory cytokines, including IL-1β, IL-6, or TNF-α have been found to promote bone resorption by favoring osteoclast formation [34]. In the present study, treatment of osteoblast-like cells with EVOO phenolic extracts reduced their expression of CD54, CD80, and HLA-DR antigens. This decrease was not related to the presence of proinflammatory cytokines or LPS but was associated with the presence of growth factors that promote osteoblast differentiation or maturation [22,24].

All of the EVOO extracts assayed *in vitro* produced a major and significant increase in ALP activity, suggesting a favorable effect on the maturation of these osteoblast-like cells. Among the four EVOOs studied, the greatest increase was observed with Picual, the variety with the highest phenolic compound content [20]. Extracts from the other three varieties also produced significant increases in ALP activity with respect to controls.

In a murine osteoblast model, Hagiwara et al [10] demonstrated that collagen proliferation and production and ALP activity were increased in MC3T3-E1 cells treated with hydroxytyrosol, tyrosol, and oleuropein, the main phenolic compounds in olive oil. In a study of old ovariectomized rats, Saleh and Saleh [35] found that 12-week treatment with EVOO (1 mL/kg body weight) significantly prevented bone thickness reduction as assessed by histology findings and ALP activity, and a reduction in their oxidative stress may have played a role.

Phagocytic capacity was observed in a high percentage of MG63 cells but was reported in only 30% of primary cultures of human osteoblasts from bone samples, and this capacity appears to be related to the degree of cell maturation [23,27]. The phagocytic capacity of MG63 cells was reduced by treatment with the phenolic EVOO extracts, supporting their beneficial effect on osteoblast maturation.

Our *in vitro* data would be related to the results observed in human studies, where it has been described that diets enriched with olive oil increase osteocalcin in serum and the concentrations of P1NP, a bone formation marker [8,36]. However, further investigations are required to have a deeper knowledge about the benefit of phenolic extracts of olive oil in bone tissue.

In conclusion, these findings suggest that phenolic extracts of EVOO contribute to osteoblast maturation, increasing ALP activity and reducing phagocytic capacity and the expression of CD54, CD80, and HLA-DR antigens. Among the four varieties studied, the greatest effects were observed with Picual.

## Supporting information

S1 Table Data for alkaline phosphatase activity, Fig 1. Relation between ALP/Proteins after treatment with phenolic extracts using Bradford´s method.
(PDF)

S2 Table Data for alkaline phosphatase activity, Fig 1. Mean, standard deviation and p value information after treatment with phenolic extracts *vs* control.
(PDF)

S3 Table Data for phenotype and phagocytosis, Figs 2 and 3. Percentage expression of antigens CD54, CD80, CD86 and HLA-DR and phagocytosis after treatment with olive oil phenolic extracts.
(PDF)

S4 Table Data for phenotype and phagocytosis, Figs 2 and 3. Mean, standard deviation and p value information after treatment with phenolic extracts *vs* control.
(PDF)
