# Supplementary material for: Effect of phenolic extracts from different extra-virgin olive oil varieties on osteoblast-like cells
Source: PLoS One. 2018 Apr 26;13(4):e0196530. doi: 10.1371/journal.pone.0196530 (PMC5919649; doi:10.1371/journal.pone.0196530)
Supplement: S1 Table — Relation between ALP/Proteins after treatment with phenolic extracts using Bradford´s method. (PDF) [file pone.0196530.s001.pdf]

S1 Table. Data for alkaline phosphatase activity, Fig 1. Relation between ALP/Proteins after treatment with phenolic extracts using Bradford´s method.

	ALP	PROTEINS	ALP/PROTEINS
**Control**	0,0633	0,555622129	0,113898403
**Control**	0,0808	0,738366287	0,109483752
**Control**	0,0826	0,757339724	0,109058819
**Picual 10^{-6}M**	0,2845	0,831236269	0,342228717
**Picual 10^{-6}M**	0,3222	0,721390054	0,446659049
**Picual 10^{-6}M**	0,2704	0,768324346	0,351972721
**Hojiblanca 10^{-6}M**	0,1230	0,847213901	0,145146633
**Hojiblanca 10^{-6}M**	0,2116	0,735370481	0,287774906
**Hojiblanca 10^{-6}M**	0,1835	0,784301977	0,234009035
**Picudo 10^{-6}M**	0,1493	0,736369083	0,202754579
**Picudo 10^{-6}M**	0,1195	0,720391452	0,165825562
**Picudo 10^{-6}M**	0,2230	0,545636109	0,408755363
**Arbequina 10^{-6}M**	0,2064	0,536648692	0,38452489
**Arbequina 10^{-6}M**	0,1853	0,561613741	0,329922862
**Arbequina 10^{-6}M**	0,1967	0,560615139	0,350864103
