# Supplementary material for: Effect of phenolic extracts from different extra-virgin olive oil varieties on osteoblast-like cells
Source: PLoS One. 2018 Apr 26;13(4):e0196530. doi: 10.1371/journal.pone.0196530 (PMC5919649; doi:10.1371/journal.pone.0196530)
Supplement: S2 Table — Mean, standard deviation and p value information after treatment with phenolic extracts vs control. (PDF) [file pone.0196530.s002.pdf]

S2 Table. Data for alkaline phosphatase activity, Fig 1. Mean, Standard deviation and p value information after treatment with phenolic extracts *vs* control.

	**Mean**	**Standard deviation**	**p value**
**Control**	0,110	0,002	-
**Picual 10^{-6}M**	0,380	0,057	0.015
**Hojiblanca 10^{-6}M**	0,255	0,028	0.012
**Picudo 10^{-6}M**	0,322	0,075	0.04
**Arbequina 10^{-6}M**	0.355	0.027	0.000
