# Supplementary material for: Effect of phenolic extracts from different extra-virgin olive oil varieties on osteoblast-like cells
Source: PLoS One. 2018 Apr 26;13(4):e0196530. doi: 10.1371/journal.pone.0196530 (PMC5919649; doi:10.1371/journal.pone.0196530)
Supplement: S3 Table — Percentage expression of antigens CD54, CD80, CD86 and HLA-DR and phagocytosis after treatment with olive oil phenolic extracts. (PDF) [file pone.0196530.s003.pdf]

S3 Table. Data for phenotype and phagocytosis, Figs 2 and 3. Percentage expression of antigens CD54, CD80, CD86 and HLA-DR and phagocytosis after treatment with olive oil phenolic extracts.

	**CD54**	**CD80**	**CD86**	**HLA-DR**	**Latex**
**Control**	75,7	21,6	13	5,1	98
**Control**	74,6	19,7	13,6	4,9	82,7
**Control**	75,4	18,3	13,3	5,2	98,8
**Mean**	**75,23**	**19,87**	**13,30**	**5,07**	**93,17**
**Picual 10^{-6}M**	61,9	4	9,7	2,4	35,6
**Picual 10^{-6}M**	61	5,2	11,9	2,4	35,4
**Picual 10^{-6}M**	62,9	4,4	10,6	3,5	32,8
**Mean**	**61,93**	**4,53**	**10,73**	**2,77**	**34,60**
**Hojiblanca 10^{-6}M**	57,8	8,1	13,9	4,6	45,2
**Hojiblanca 10^{-6}M**	56,3	9	14,6	4,4	44,3
**Hojiblanca 10^{-6}M**	57,1	8,4	12,2	4,5	46,4
**Mean**	**57,07**	**8,50**	**13,57**	**4,50**	**45,30**
**Picudo 10^{-6}M**	51,3	10,7	6,7	3,5	33,2
**Picudo 10^{-6}M**	47,3	12,6	7,8	4,5	37
**Picudo 10^{-6}M**	50	5,7	6,3	3,5	37,4
**Mean**	**49,53**	**9,67**	**6,93**	**3,83**	**35,87**
**Arbequina 10^{-6}M**	38,8	2,9	14,3	4,4	39,4
**Arbequina 10^{-6}M**	36,9	2,6	13,4	3,2	39,1
**Arbequina 10^{-6}M**	36,4	2,9	12,2	3,4	39
**Mean**	**37,37**	**2,80**	**13,30**	**3,67**	**39,17**
