# Supplementary material for: Effect of phenolic extracts from different extra-virgin olive oil varieties on osteoblast-like cells
Source: PLoS One. 2018 Apr 26;13(4):e0196530. doi: 10.1371/journal.pone.0196530 (PMC5919649; doi:10.1371/journal.pone.0196530)
Supplement: S4 Table — Mean, standard deviation and p value information after treatment with phenolic extracts vs control. (PDF) [file pone.0196530.s004.pdf]

S4 Table. Data for phenotype and phagocytosis, Figs 2 and 3. Mean, standard deviation and p value information after treatment with phenolic extracts *vs* control.

	CD54		CD80		CD86		HLA-DR		Latex	
	**Mean sd**	**p value**	**Mean sd**	**p value**	**Mean sd**	**p value**	**Mean sd**	**p value**	**Mean sd**	**p value**
**Control**	75,23 (0,56)	-	19,86 (1,65)		13,30 (0,30)	-	5,06 (0,15)	-	93,16 (9,07)	-
**Picual 10^{-6}M**	61,96 (0,95)	0,000	4,53 (0,61)	0,000	10,73 (1,10)	0,048	2,76 (0,63)	0,02	34,60 (1,56)	0,007
**Hojiblanca10^{-6}M**	57,06 (0,75)	0,000	8,50 (0,45)	0,004	13,56 (1,23)	0,73	4,50 (0,10)	0,006	45,30 (1,05)	0,011
**Picudo10^{-6}M**	49,53 (2,04)	0,000	9,66 (3,56)	0,023	6,93 (0,77)	0,000	3,83 (0,57)	0,05	35,86 (2,31)	0,006
**Arbequina 10^{-6}M**	37,36 (1,26)	0,000	2,80 (0,17)	0,003	12,96 (1,15)	0,65	3,66 (0,64)	0,021	39,16 (0,20)	0,009
